# The complete mitochondrial genome of *Sarcophila rasnitzyni* (Diptera: Sarcophagidae)

**DOI:** 10.1080/23802359.2021.1955634

**Published:** 2021-09-13

**Authors:** Jingyuan Ma, Huixiao Yu, Zhouru Li, Wenjiang Yin, Hongxing Cai

**Affiliations:** Department of Forensic Science, Xuzhou Medical University, Xuzhou, China

**Keywords:** Mitochondrial genome, *Sarcophila mongolica*, phylogenetic analysis

## Abstract

*Sarcophila rasnitzyni* Rohdendorf and Verves, 1985 (Diptera: Sarcophagidae) is of potential significance in medicine and epidemiology. In this study, we present the mitochondrial genome of *S. rasnitzyni*. The full length of the mitochondrial genome is 15,321 bp (GenBank accession no. MW592359), and 13 protein-coding genes (PCGs), two ribosomal RNAs (rRNAs), 22 transfer RNAs (tRNAs), and a non-coding control region were identified. Nucleotide composition is A 38.0%, G 9.9%, C 14.9%, T 37.2%, respectively. It reveals a strong A + T bias (75.2%). Phylogenetic analysis indicates that the species-level relationship between *S. rasnitzyni* and *S. mongolica* closely clusters together, and separates clearly from the rest of species. This study provides important genetic data for further enriching our understanding of phylogenetic relationship of sarcophagids species.

Sarcophagid flies (known as flesh flies) can lead to global health concerns as carriers of various pathogenic micro-organisms, additionally, they usually colonized on the decomposed corpses and constitute a part of the insect faunal succession representing important destruction stage responsible for the essential decomposition (Ren et al. 2018). *Sarcophila rasnitzyni* Rohdendorf and Verves, 1985 (Diptera: Sarcophagidae) mainly distributed in China (Beijing, Heilongjiang, Neimenggu, Qinghai, Xinjiang), Mongolia and Russia (East Siberia) (Pape 1996; Xu and Zhao 1996). The adult of *S. rasnitzyni* colonized on the decomposed carcasses (Xu and Zhao 1996). Except this species, only one species of *Sarcophila* genus (*Sarcophila mongolica* Chao and Zhang) has been recorded in China (Xu and Zhao 1996). Here, we present the complete mitochondrial genome (mitogenome) of *S. rasnitzyni*, which would further enrich our understanding of the phylogenetic relationship of *Sarcophila* genus (Cameron 2014).

In this study, the adult specimens of *S. rasnitzyni* were trapped by pig lung in June 2020, Beijing (40°22′N, 166°23′ E), China. All the specimens were identified by traditional morphological methods (Xu and Zhao 1996), and then soaked in 95% alcohol and placed in −20 °C (Xuzhou Medical University, Jiangsu, China) with a unique code (CSU20210201). Total DNA was extracted from thoracic muscle tissues of an adult specimen using QIANamp Micro DNA Kit (Qiangen Biotech Co., Ltd, Germany). The mitogenome sequencing of *S. rasnitzyni* was performed on an Illumina HiSeq 2500 Platform (pared-end 150 bp), and then assembled based on Illumina short reads with MitoZ v2.3 (Meng et al. 2019). The rough boundaries of all genes was annotated by MITOS2 Web Server (http://mitos2.bioinf.uni-leipzig.de/index.py) under the invertebrate mitochondrial code (Bernt et al. 2013). In order to confirm the correctness of gene boundaries, 13 protein coding genes (PCGs) were aligned with published mitogenomes of flesh flies using Muscle (codons) as implemented in MEGA X (Kumar et al. 2018). Two ribosomal RNAs (rRNAs) were aligned by the Q-INSi method as implemented in MAFFT v7.263 (Katoh and Standley 2016). 22 transfer RNAs (tRNAs) were predicted by tRNAscan-SE Search Server v1.21 (Lowe and Chan 2016) and verified by aligning with other dipteran insects.

In this study, the total length of the mitogenome of *S. rasnitzyni* is 15,321 bp (GenBank accession no. MW592359). It obtains 13 PCGs, two rRNAs, 22 tRNAs, and a non-coding control region. The gene arrangement is consistent with that of ancestral metazoan (Cameron 2014). All PCGs initiate with a typical start codon (ATN), except that *cox1* starts with TCG. Most PCGs end with the termination codons TAA/TAG, in addition that four genes (*cox1*, *cox2*, *nad4* and *nad5*) are terminated with T. Nucleotide composition of *S. rasnitzyni* is A 38.0%, G 9.9%, C 14.9%, T 37.2%. It indicates a highly A + T bias (75.2%). Additionally, the size of overlap regions was examined, varying from 1 to 9 bp. The longest intergenic spacers (18 bp) are situated between trnE and trnF.

Furthermore, phylogenetic tree was constructed with *S. rasnitzyni* and other 14 sarcophagids species based on the 13 PCGs by maximum likelihood (ML) method with the GTR + G + I model as implemented in IQ-TREE v.1.6.12 (Nguyen et al. 2015). *Calliphora vomitoria* (Diptera: Calliphoridae) was used as an outgroup (Figure 1). In the phylogenetic tree, the species-level relationship between *S. rasnitzyni* and *S. mongolica* closely clusters together, and separates clearly from the rest of species. The branches are well supported. This study provides important mitochondrial data for further enriching our understanding of phylogenetic relationship of sarcophagids species.

**Figure 1.**
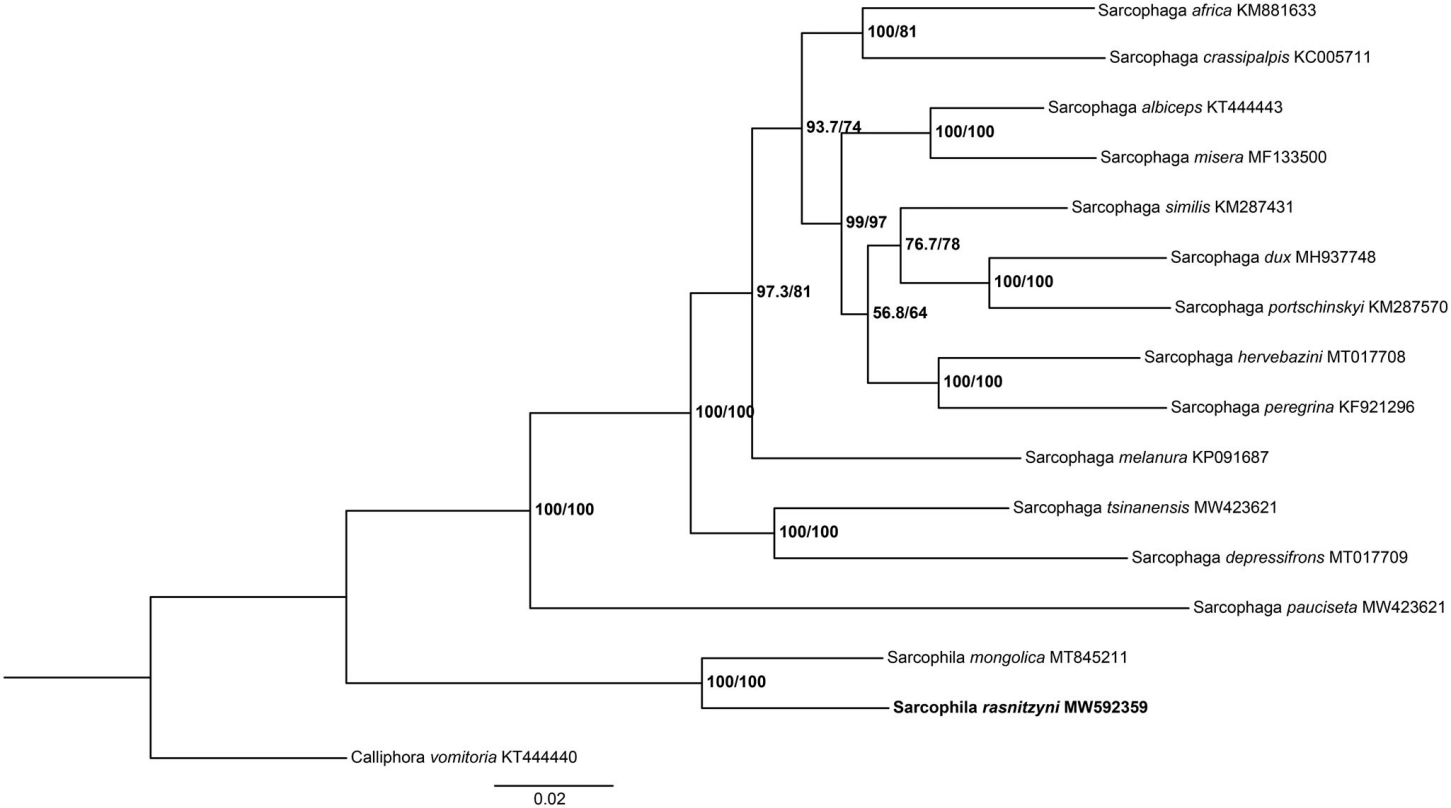
Phylogenetic tree of *S. rasnitzyni* with 14 sarcophagids species based on 13 PCGs by maximum likelihood (ML) method. *Calliphora vomitoria* was selected as an outgroup.

## Data Availability

Mitogenome data supporting this study are openly available in GenBank at: https://www.ncbi.nlm.nih.gov/nuccore/MW592359. Associated BioProject, SRA, and BioSample accession numbers are https://dataview.ncbi.nlm.nih.gov/object/PRJNA700699, https://www.ncbi.nlm.nih.gov/sra/SRR13757193, and SAMN17838720, respectively.
